# Supervised Machine Learning-Based Models for Predicting Raised Blood Sugar

**DOI:** 10.3390/ijerph21070840

**Published:** 2024-06-27

**Authors:** Marwa Mustafa Owess, Amani Yousef Owda, Majdi Owda, Salwa Massad

**Affiliations:** 1 Department of Natural, Engineering, and Technology Sciences, Arab American University, Ramallah P600, Palestine; m.owess@student.aaup.edu; 2 The World Health Organization, Jerusalem P.O. Box 54812, Palestine; salwamassad@gmail.com; 3 Faculty of Data Science, UNESCO Chair in Data Science for Sustainable Development, Arab American University, Ramallah P600, Palestine; majdi.owda@aaup.edu

**Keywords:** raised blood sugar, diabetes, machine learning, prediction, classification

## Abstract

Raised blood sugar (hyperglycemia) is considered a strong indicator of prediabetes or diabetes mellitus. Diabetes mellitus is one of the most common non-communicable diseases (NCDs) affecting the adult population. Recently, the prevalence of diabetes has been increasing at a faster rate, especially in developing countries. The primary concern associated with diabetes is the potential for serious health complications to occur if it is not diagnosed early. Therefore, timely detection and screening of diabetes is considered a crucial factor in treating and controlling the disease. Population screening for raised blood sugar aims to identify individuals at risk before symptoms appear, enabling timely intervention and potentially improved health outcomes. However, implementing large-scale screening programs can be expensive, requiring testing, follow-up, and management resources, potentially straining healthcare systems. Given the above facts, this paper presents supervised machine-learning models to detect and predict raised blood sugar. The proposed raised blood sugar models utilize diabetes-related risk factors including age, body mass index (BMI), eating habits, physical activity, prevalence of other diseases, and fasting blood sugar obtained from the dataset of the STEPwise approach to NCD risk factor study collected from adults in the Palestinian community. The diabetes risk factor obtained from the STEPS dataset was used as input for building the prediction model that was trained using various types of supervised learning classification algorithms including random forest, decision tree, Adaboost, XGBoost, bagging decision trees, and multi-layer perceptron (MLP). Based on the experimental results, the raised blood sugar models demonstrated optimal performance when implemented with a random forest classifier, yielding an accuracy of 98.4%. Followed by the bagging decision trees, XGBoost, MLP, AdaBoost, and decision tree with an accuracy of 97.4%, 96.4%, 96.3%, 95.2%, and 94.8%, respectively.

## 1. Introduction

Since 1980, the number of people with diabetes has skyrocketed, rising from 108 million to a staggering 422 million by 2014, with a disproportionate increase observed in low- and middle-income countries [1]. This alarming rise makes diabetes a major global health concern, not only leading to blindness, kidney failure, heart attacks, strokes, and amputations but also contributing to a significant rise in mortality, with an estimated 2 million deaths attributed to diabetes and its related kidney complications in 2019 alone [1].

**Figure 1:**
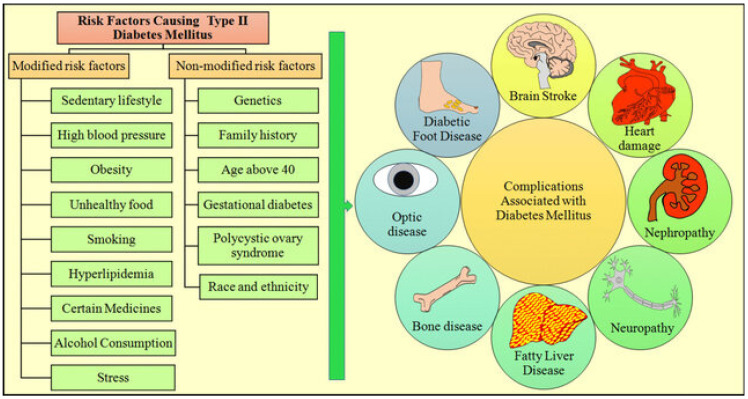
Type 2 diabetes mellitus risk factors and complications [13].

The raised glucose level in the bloodstream is considered a primary characteristic of diabetes. That develops when the production of insulin hormones by the pancreas gland is insufficient enough to effectively regulate glucose levels resulting from the breakdown of the food we consume as a source of energy for humans [2]. The prevalent symptoms experienced among diabetic patients often include excessive thirst and hunger, frequent urination, fatigue, blurred vision, and weight loss [3]. Diabetes is divided broadly into two major types, type 1 diabetes and type 2 diabetes. Type 1 diabetes is characterized as a severe inadequacy or absence of releasing insulin by the pancreas due to an unknown disorder in the immune system, it affects people at a younger age more often, and it also can affect children. The primary treatment method for type 1 diabetes is insulin therapy. Type 2 diabetes is characterized by insulin resistance in which the body has insulin but does not utilize it well to regulate the blood sugar levels [4]. This type is considered the most common one among diabetes patients [5]. Recently, the incidence of type 2 diabetes has become alarmingly high, which is attributed to several reasons related to risk factors associated with diabetes, such as obesity, poor eating habits, lack of physical activity, smoking, and alcohol consumption [6]. As per the statistics published by the International Diabetes Federation in 2021, it is reported that over half a billion adults have been diagnosed with diabetes [7]. The main burden associated with diabetes is that it can lead to developing risky health complications if individuals with diabetes do not receive the proper care, treatment, and management of the related risk factors [8]. Some of the most commonly identified complications among diabetic patients included heart diseases, kidney failure, neuropathy, teeth and gum diseases, peripheral arterial disease, foot problems, retinopathy, blindness, and cerebrovascular diseases [9,10,11]. As a result of these health complications associated with diabetes, it was recognized by the World Health Organization (WHO) as one of the top ten leading causes of death worldwide [12]. However, the consequences of diabetes can be mitigated through early diagnosis and effective management [13]. Figure 1 illustrates some risk factors and complications associated with diabetes Type 2.

The medical methods typically used to diagnose diabetes include the fasting blood sugar test, hemoglobin A1C (HbA1C) test, or oral glucose tolerance test (OGTT), each type of these tests has a different procedure and threshold for diagnosing diabetes, for the HbA1C test, the threshold is 6.5% or higher, in the case of OGTT the threshold is 200 mg/dL or above [14,15]. Regarding the fasting blood sugar test, the normal range is between 70 and 99 milligrams per deciliter (mg/dL), readings between 100 and 125 mg/dL are defined as impaired fasting glucose and might be an indication of the condition of prediabetes, while when the fasting blood sugar measures 126 mg/dL or higher, it is recognized as raised blood sugar which may indicate diabetes if the results of the fasting blood sugar for two separate tests exceed this threshold [16].

With the aim of early detection of diabetes to avoid its potential complications, machine learning can be employed to meet this purpose, as machine learning techniques have proven their efficiency in constructing powerful accurate predictive models, serving as assisting tools for healthcare professionals in detecting diseases specifically [17,18,19].

In this context, this paper presents an innovative model for predicting raised blood glucose among individuals as a screening tool for diabetes, by utilizing machine learning algorithms and applying them to a dataset that was collected to study the risk factors of non-communicable diseases (NCDs), focusing on raised blood glucose, raised blood pressure, and high blood cholesterol diseases.

The key contribution of the raised blood sugar prediction model proposed in this paper is its training on a unique first-hand and comprehensive dataset, considering both the number of features and observations, which combine all the relevant risk factors of diabetes including gender, age, BMI, smoking status, physical activity level, alcohol consumption level, stress level, sugar and salt intake, medical history, blood pressure, blood lipids, and physical measurement variables, which was used for the first time for modeling purposes, achieving significantly higher accuracy when compared to previous work presented in this field. It addressed the challenges faced by prior proposed models that were demonstrated on frequently used datasets containing a small set of feature issues, in addition to data quality issues [20,21]. Moreover, the proposed models can serve as a supportive tool to aid in screening for diabetes, by identifying adults with raised blood sugar levels, facilitating referrals for further diagnosis, and enabling early detection. Notably, it offers the advantage of reducing the time and costs used in other standard approaches for diabetes diagnosis, especially in developing countries, as the target feature in this study is based on the readings of fasting blood sugar test, which is considered a low-cost, accessible, and effective method for diabetes screening. The results generated by the proposed models can prompt individuals to take necessary preventive measures and manage risk factors associated with diabetes, including adjusting their lifestyle and monitoring their health. As well as this, it identifies potential cases for undergoing additional tests for timely detection and treatment.

This section presented an overview of the raised blood sugar and the condition of diabetes, the following sections in this paper are organized as follows: Section 2 introduces a literature review of using machine learning to predict glucose metabolism disorders and discusses the related studies in this field. Section 3 illustrates the methodology used to build the raised blood sugar prediction model, and Section 4 discusses the experimental results. Section 5 presents the conclusion and the plans for future work.

## 2. Literature Review

Several studies have presented data-driven models for predicting glucose metabolism disorders by utilizing various machine learning algorithms and techniques [22], including supervised learning algorithms, neural networks, or image processing techniques [23,24]. These studies that are related to glucose metabolism disorders prediction involved either diabetes, prediabetes, hyperglycemia, or raised blood sugar. The author of [25] presented a model for predicting diabetes by using a dataset collected from native American Indian females (PIDD dataset) [26]. The presented model utilized artificial neural networks (ANN), random forest (RF), and K-means clustering algorithms. The ANN-based model achieved the best results among the three implementations with accuracy of 75.7%, also this study aimed to characterize the relationship between the significant risk factors responsible for causing diabetes using Apriori association rules, which showed that diabetes is strongly associated with body mass index and glucose level. Another comparative study in [27] was demonstrated on the PIDD dataset for predicting diabetes using a set of machine-learning algorithms including Support Vector Machine (SVM), decision tree (DT), K Nearest Neighbor (KNN), random forest, naïve Bayes (NB), AdaBoost, logistic regression (LR), and ANN for building the models, each algorithm was tested using two methods, once by splitting into training and testing datasets and another time using k-fold cross validation technique. The model built using ANN had the highest accuracy score of 88.6%. The authors of [28] presented a comparative study for building a diabetes classification model using different machine learning classification algorithms including random forest, SVM, KNN, and decision tree J48. The study utilized the UCI diabetes public dataset consisting of variables of diabetes symptoms [29], although the experimental results of the proposed model were excellent in terms of accuracy it still needs more testing and investigation by demonstrating to a more challenging dataset since it was applied to a dataset of a minimal set of features having binary classifications only.

The study in [30] presented a machine learning-based diabetes detection model that can be used as a clinical decision support system for assisting healthcare workers in diagnosing diabetes cases, the proposed model was implemented using a set of machine learning algorithms including random forest, SVM, and deep convolutional neural networks (CNN), the diabetes detection model achieved the best results when utilized using random forest with an accuracy of 83.6% model, also the accuracy was 76.81% using deep CNN, and 65.38% using SVM. The authors in [31] proposed a deep learning model for diabetes prediction using artificial neural networks (ANN), naïve Bayes, decision tree, and deep learning (DL) algorithms, the model was applied to the American Indian females’ diabetes public dataset and resulted in high accuracy of 98.07%. However, the study did not discuss the model performance in case of using a low-quality dataset that might contain missing values or unbalanced data. The study in [32] proposed a hybrid prediction model for predicting type 2 diabetes, which is based on using K-means and logistic regression techniques. Using the K-means algorithm in this model helped eliminate incorrect clustered data in the preprocessing stage, before applying the logistic regression algorithm for performing the classification. The demonstrated diabetes prediction model achieved an excellent prediction accuracy of 93.9% in comparison with previous works applied to the same dataset. The authors in [33] presented a comparative study for a different set of machine learning algorithms that were used in building models for predicting diabetes based on the related risk factors including age, gender, BMI, family history of diabetes, marital status, education level, stress, sleep, physical activity, diet, salt consumption, and drinking coffee. The study focused on comparing the performance of ANNs, decision tree, and logistic regression machine learning algorithms in building the diabetes prediction models. Using the decision tree algorithm in the diabetes prediction model achieved the best performance results with an accuracy of 77.87% while using the ANN resulted in the lowest accuracy. The proposed model was applied to a balanced dataset collected from Chinese adults including observations from both diabetes patients and normal adults not diagnosed with diabetes. The authors of [34] presented a machine learning model for predicting diabetes and cardiovascular disease using a dataset obtained from the National Health and Nutrition Examination Survey (NHANES) [35]. The NHANES dataset was collected by an ongoing study that includes personal interviews, physical examinations, and lab test results. The authors divided the collected NHANES dataset into two subsets, one having the diagnosis of diabetes reported by participants. The second subset included subjects with fasting blood sugar (FBS) greater or equal to 126 mg/dL and diabetes pre-diagnosis not reported by participants, to be considered diabetic patients. The main features used in this study are physical characteristics (age, waist size, leg length, etc.), dietary intake (sodium, fiber, caffeine intake), demographics (ethnicity and income), and laboratory test results (HDL, LDL, cholesterol, urine). The proposed diabetes prediction model in that study was implemented by testing different machine learning classification algorithms such as logistic regression, SVM, random forest, and gradient boosting trees on the two subsets, once without including laboratory variables as the input for the models and another time using laboratory tests. The tested algorithms were combined to implement a weighted ensemble to enhance the achieved performances. The testing results using the five models of the four different cases of the dataset achieved the best performance using the XGBoost algorithm, when it was applied to the subset that includes the lab test results and reported diabetes diagnosis as the target feature. Another study was presented and utilized the NHANES dataset for predicting prediabetes [36]. The proposed prediabetes models employed several machine learning algorithms including random forest, AdaBoost, LogitBoost, logistic regression, J48, naïve Bayes, PART, sequential minimal optimization algorithm (SMO) SVM, and instance-based learner (IBk). The used features for training the prediabetes prediction models were BMI, family history of diabetes, race, hypertension patient, and total cholesterol, while the target categorical yes/no feature was derived from any one of the available tests of either FBS, HbA1c, or 2 h postprandial glucose test (2hrPG). The naïve Bayes prediabetes prediction model achieved the best positive predictive value of 74.5%. The work in [37] presented machine learning-based models for predicting the risk of glucose metabolism disorders using a private dataset collected from employees working in a certain Japanese corporation who underwent annual medical examinations. The proposed glucose metabolism disorders prediction models were based mainly on using XGBoost and logistic regression classifiers, which were trained on a dataset that includes age, sex, BMI, systolic and diastolic blood pressure, triglyceride, HDL, LDL, creatinine, immunoreactive insulin (IRI), 1 h and 2 h plasma glucose (PG), and total cholesterol. The OGTT, FBS, and HbA1C test results were used to determine the class of the target feature that represents whether an observation belongs to the group at risk of glucose metabolism disorders or not. The model achieved the best performance using the XGBoost algorithm.

Table 1 summarizes the main findings of the previous works and the literature proposed to predict different types of glucose metabolism disorders, including the used features, the utilized algorithms, the best-performing model, and its best outcome result.

By investigating the related works and literature, it was found that several previous studies have been proposed within the scope of predicting glycemic conditions. Those works mainly relied on either the diabetes diagnosis which is self-reported by the patients [25,27,28,30,31,32,33] or by deriving the classification of diabetes, prediabetes, or glucose metabolism disorders using either the fasting blood sugar or HbA1C or OGTT [33,34,36,37]. Many of these studies commonly referred to the term diabetes in naming their models [25,27,28,30,31,32,33], while others titled their studies using the term glucose metabolism disorders [37]. In addition to other models that targeted the prediction of prediabetes [33,36]. In this study, the title of the proposed work used the term raised blood sugar specifically, which is identified by the level of fasting blood sugar higher or equal to 126 mg/dL, to be more accurate in the use of the model as a screening tool for detecting raised blood sugar levels that might be suspected cases of diabetes. The aim is to recommend monitoring blood sugar levels by taking repeated readings and facilitating referral of detected cases for diabetes diagnosis, considering the sensitivity of models proposed to support the health and medical domain.

The raised blood sugar prediction models proposed in this paper are trained on a unique, comprehensive dataset that includes a wide range of relevant risk factors such as gender, age, BMI, smoking status, physical activity, alcohol consumption, stress level, dietary intake, medical history, blood pressure, blood lipids, and physical measurements. This dataset, specifically collected to study NCD risk factors, allowed for significantly higher accuracy compared to previous studies. The proposed models can serve as supportive tools for screening diabetes, identifying adults with elevated blood sugar levels, and facilitating early detection and referrals. Additionally, these models offer a cost-effective alternative to standard screening approaches, especially in developing countries, by utilizing accessible methods like fasting blood sugar tests. The models’ results can prompt individuals to take preventive measures and manage risk factors, thereby improving public health by reducing premature mortality rates and healthcare expenditures associated with high blood sugar and diabetes.

## 3. Methodology

This section illustrates the phases of the proposed raised blood sugar prediction models, including the description of the used dataset, the data cleaning and preparation, the exploratory data analysis, data preprocessing, and proposed machine learning-based models for building the raised blood sugar prediction using random forest, decision tree, XGBoost, Adaboost, bagging decision trees, and multi-layer perceptron (MLP) classification algorithms.

**Figure 2:**
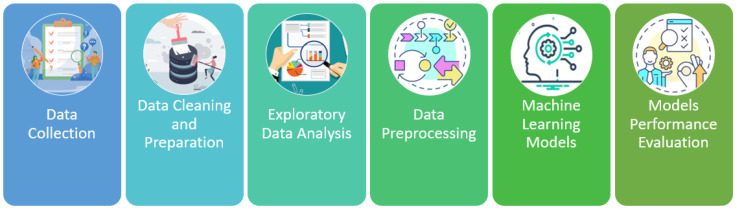
Main phases of the raised blood sugar detection models.

Figure 2 depicts the main phases of the implemented raised blood sugar (RBS) detection models. The next subsections within this section will provide detailed insights into these six phases and their inside components utilized in constructing the proposed models for raised blood sugar detection.

### 3.1. Data Collection and Dataset Description

The dataset utilized in this work for constructing the raised blood sugar prediction models was obtained from surveys conducted as part of the STEPS study (STEPwise approach to NCD risk factor surveillance) [38]. The STEPS study, standardized by the World Health Organization, focuses on assessing the risk factors of NCDs and their prevalence across different countries. As the name of the study indicates, it consists of three levels of risk factor assessment, the first level is a questionnaire, the second is for physical measures, and the last step is biochemical measures. The STEPS study was conducted by the Palestinian National Institute of Public Health in collaboration with the Palestinian Ministry of Health in Palestine in 2022.

The STEPS dataset was collected by conducting interviews with participants, encompassing demographic, social, economic, smoking, alcohol consumption, eating habits, health history, sleeping habits, physical activity levels, and mental health questions. Physical measurements were also recorded, including blood pressure, heart rate, weight, height, and BMI calculations. Additionally, the dataset includes laboratory test results for fasting blood glucose, HDL, total cholesterol, and triglyceride levels. It consists of more than 5 thousand records and around 130 variables.

The target population of the collected dataset is Palestinian adults aged between 18 and 69 of both sexes who have been living in Palestine for at least 12 months. The used sampling approach in collecting this dataset is three-stage stratified cluster sampling to select a random sample of adults from 525 enumeration areas in the West Bank and the Gaza Strip, then selecting 11 households from each enumeration area. The first stage was selecting the enumeration areas, the second stage was selecting households from each enumeration area by using blind maps provided by the Palestinian Central Bureau of Statistics and the Kish table sampling method, and the third stage was selecting a participant from the households. The total sample size is 5775 participants. The data collection team consisted of two members, the first member is a field worker responsible for conducting personal interviews with participants that aim to collect demographic, socio-economic, and personal lifestyle information. The second one is a nurse who is responsible for collecting medical history, recording the physical measurements, and performing the blood tests for participants.

It is worth pointing out that the adoption of the STEPs dataset in this study to build machine learning models represents a contemporary pattern, as there is a very limited number of studies that utilized the STEPs dataset in training machine learning-based models since the start of conducting the NCDs stepwise approach study. The STEPS Palestine 2022 dataset is owned by the Palestinian Ministry of Health and is still not available publicly.

### 3.2. Data Cleaning and Preparation

This phase is considered an important step to prepare the data for the next phases of dataset exploration and modeling as well, which might be affected due to data quality issues. This phase mainly involved steps for cleaning the STEPS dataset.

- Handling missing values: Upon exploration of the STEPS dataset, it was observed to contain some missing values. On checking for missing values, several issues were identified and addressed. Records with consent values of 2 and empty remaining variables, indicating refusal to participate, were excluded. Pregnant women’s records, which lacked physical measurements as per STEPs survey guidelines, were also dropped to maintain accuracy. Special codes used as placeholders for missing categorical values were replaced with the mode of the respective variables. Missing values in conditional variables, such as daily smoking based on current smoking, were filled with zero. For missing biochemical measurements, the issues stemmed from either lack of consent for Step 3 participation or non-adherence to fasting instructions, observations belonging to these cases were handled by dropping them from the dataset.
- Handling outliers: The STEPS dataset comprises various types of data, including data about the participant’s lifestyle, medical history, and lab test results. Some of these features contain abnormal values. A visual approach, utilizing boxplots, was employed to detect outlier values. The feedback provided by domain experts along with the research results for identifying normal ranges were used to handle the outlier values either by dropping or substitution.

### 3.3. Exploratory Data Analysis

In data-driven models, exploratory data analysis using either statistical or graphical methods plays an important role in investigating the dataset [39]. Therefore, in this study, exploratory data analysis techniques using a graphical approach were used, which provided a comprehensive understanding of every single feature in the dataset, along with detecting the different relationships between the dataset variables. The illustrated figures below show some characteristics of the used STEPS dataset, in addition to different relationships between the dataset variables.

**Figure 3:**
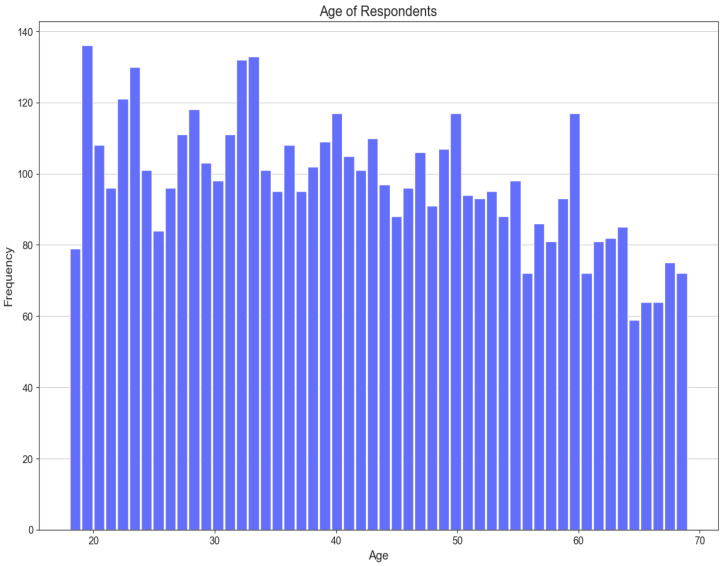
Age distribution of participants in the STEPS dataset.

As mentioned earlier, the population of the STEPS study is adults between 18 and 69 years. Figure 3 shows the frequency of participants’ ages in the STEPS dataset.

**Figure 4:**
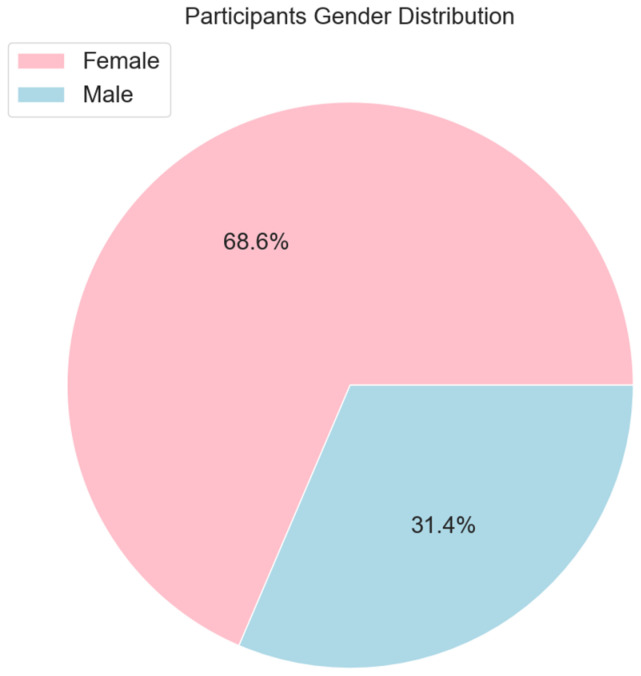
The distribution of participants in the STEPS dataset by sex.

Figure 4 shows the distribution of participants in the study by their gender. The majority of the participants are from the female group.

**Figure 5:**
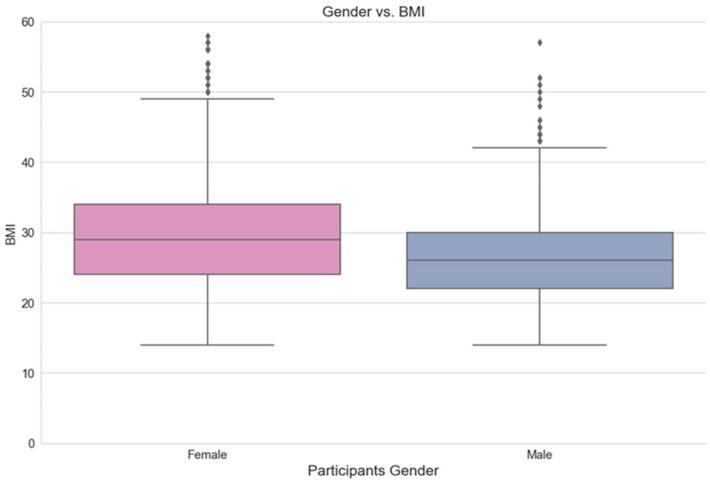
Comparison of BMI rates by participants’ gender.

Figure 5 shows a comparison between the female group and the male group of participants, in terms of their BMI. It is evident that female participants have higher BMI rates than males.

**Figure 6:**
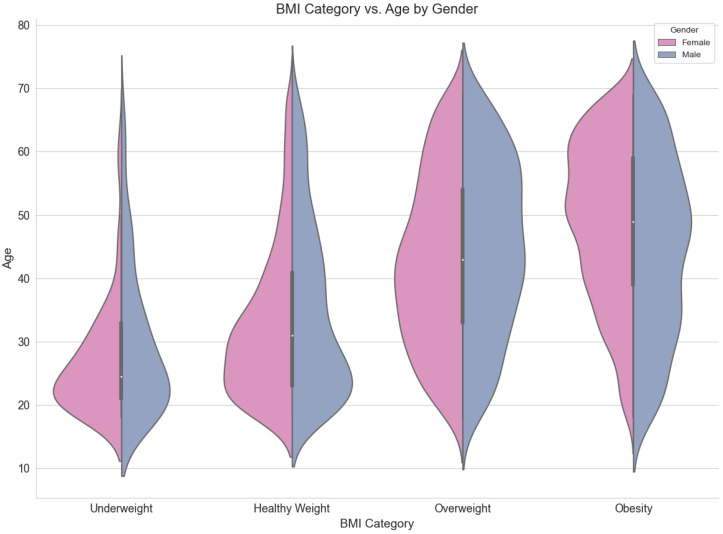
Comparison of BMI classifications by age and gender.

Figure 6 shows the distribution of the weight categories of participants by age and gender. From the graph below, it is clear that overweight and obesity are more prevalent among participants at higher ages.

**Figure 7:**
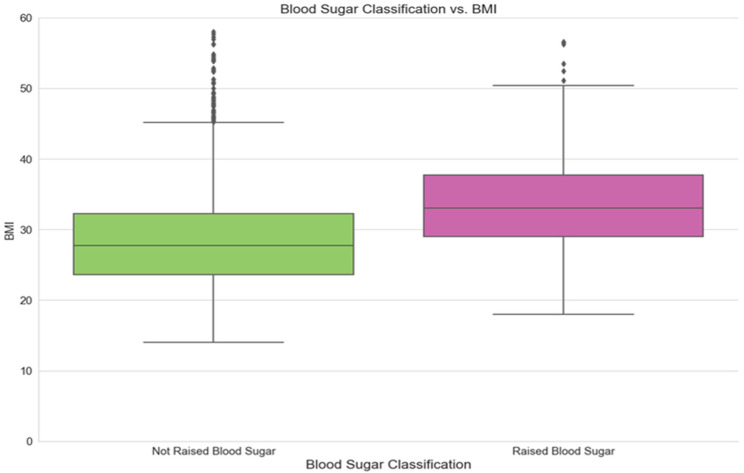
Blood sugar classifications by BMI.

Figure 7 below illustrates the relationship between the classification of the fasting blood sugar and the BMI of the participant, which shows that subjects of the raised blood sugar class have higher BMI rates. It should be noted that the term “Not Raised Blood Sugar” in all of the graphs below refers to the category of subjects that involves cases of normal and impaired blood sugar levels.

**Figure 8:**
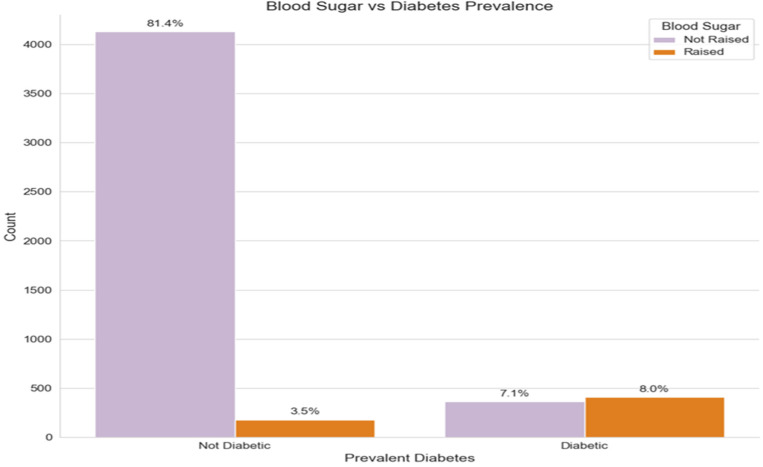
Comparison of blood sugar between diabetic and nondiabetic groups.

Figure 8 depicts the prevalence of diabetes reported by the participant through the medical history interview question “Have you ever been told by a doctor or health worker that you have diabetes”. As shown in the below figure, the detected raised blood sugar cases by the fasting blood sugar test among the diabetic group represent 8%, which indicates that diabetes among this diagnosed group is not controlled. On the other hand, in the non-diabetic group, the percentage of participants who have raised blood sugar is 3.5%, which may indicate undiagnosed cases of diabetes.

**Figure 9:**
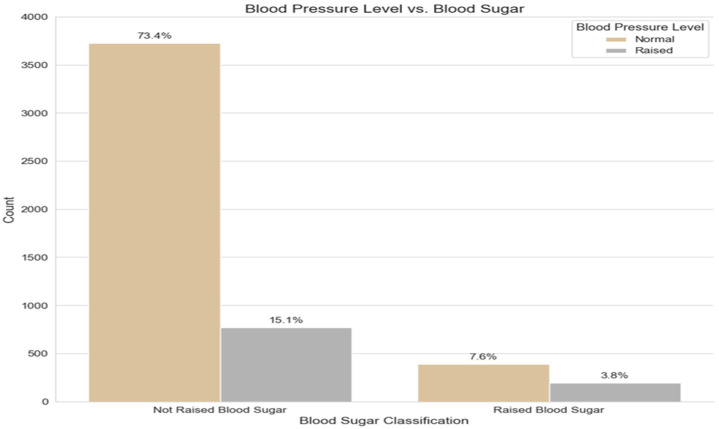
The relationship between blood pressure and blood sugar levels.

Figure 9 illustrates the relationship between the classification of the blood pressure levels measured by three separate readings and the blood sugar level among the participants in the STEPS dataset. It shows that around twice the number of cases in the raised blood pressure class also have raised blood sugar. This emphasizes the results that have been concluded by several previous studies about the strong association between hypertension and hyperglycemia conditions [40,41].

**Figure 10:**
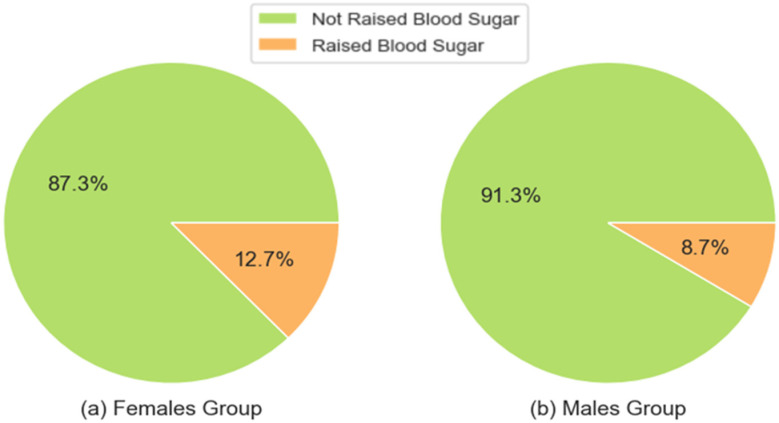
Comparison of the raised blood sugar prevalence between the female and male groups.

Figure 10 shows a comparison of the prevalence of raised blood sugar levels between the female participants group and male participants, which is higher among the women’s group than the men’s group.

**Figure 11:**
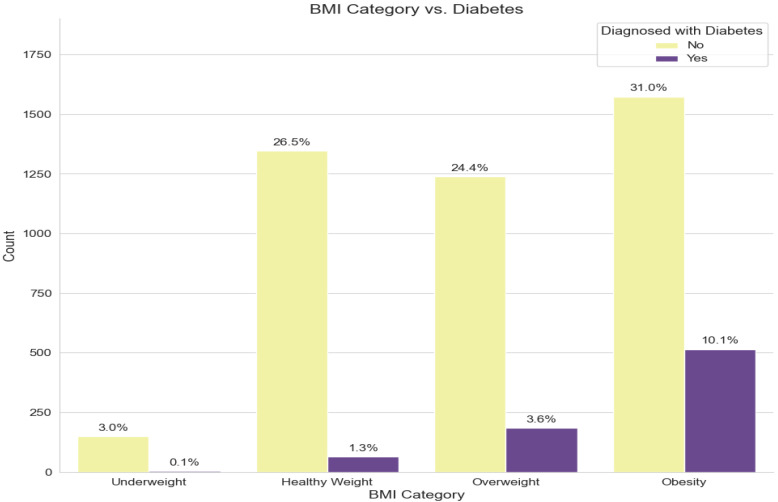
The prevalence of diabetes among participants by their BMI class.

Figure 11 shows the prevalence of reported diabetes among participants distributed by the BMI category according to their weight. In the case of participants who are classified as obese, it is obvious that around 30% are diagnosed with diabetes, for participants in the overweight class, around 15% of them are diabetic patients, while these percentages are significantly lower in the healthy weight and underweight categories. This indicates a relationship between obesity and overweight with diabetes, which is determined as a risk factor for developing diabetes [42,43,44].

**Figure 12:**
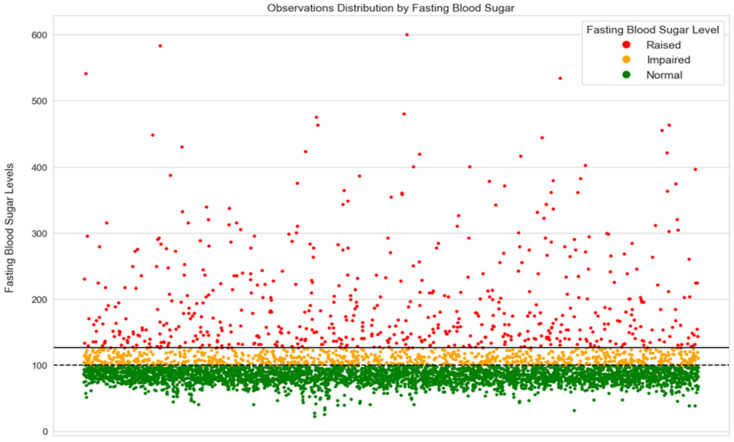
The distribution of participants by fasting blood sugar.

Figure 12 shows the distribution of the observations according to the results of the fasting blood sugar test. The green observations indicate subjects with fasting blood sugar levels less than 100 mg/dL belong to the normal class, while the observations illustrated in orange, which are located above the dashed line, represent the impaired cases who have fasting blood sugar levels between 100 mg/dL and 125 mg/dL. The observations of fasting blood sugar that are higher or equal to 126 mg/dL, which are depicted in green in the graph and situated above the continuous line that represents the threshold of raised levels, are classified as raised blood sugar cases. For the raised blood sugar cases, it is evident that the observations are sparse, indicating irregular sugar levels in blood.

### 3.4. Data Preprocessing

Preparing data is a crucial step in modeling, which is necessary to address any issues with the data before applying machine learning models. This ensures optimal results by utilizing clean and uniform data. Since data quality significantly influences the effectiveness of models, the initial step in implementing the proposed model involves preprocessing the STEPS dataset. Data preparation and preprocessing for the STEPS dataset involved the following steps.

#### 3.4.1. Determining Target Feature

The target feature in the proposed model is a binary variable derived from the fasting blood sugar reading that is available in the processed STEPS dataset, which indicates whether the participant has a raised blood sugar or not, based on the threshold identified by WHO, which determines levels of 126 mg/dL or higher of fasting blood sugar as elevated blood glucose cases [16]. The positive class in this feature reflects the raised blood sugar cases, while the negative class means not raised cases with fasting blood sugar levels less than 126 mg/dL, which involves both normal and impaired cases.

#### 3.4.2. Feature Selection

Various techniques were employed to identify the most significant features for constructing the raised blood sugar prediction model and eliminating the irrelevant features, which was concluded by several researchers and previous studies to have an impact on improving the accuracy of classification algorithms [45,46]. The involved feature selection techniques in this study include correlation matrix, chi-square, and random forest classifier feature importance.

**Figure 13:**
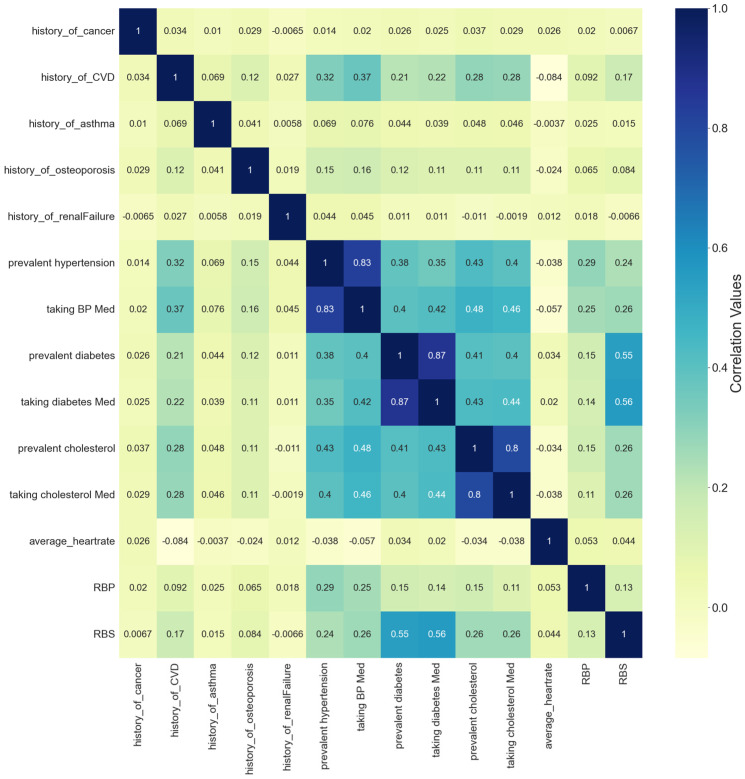
Variables correlation matrix.

**Figure 14:**
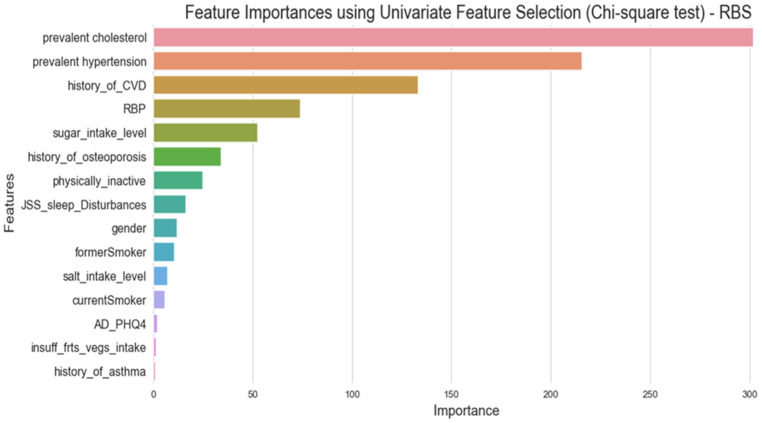
Feature importance using univariate feature selection (chi-square test).

**Figure 15:**
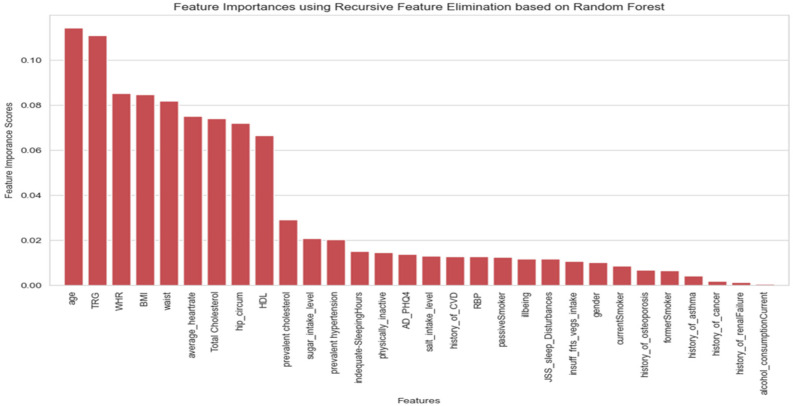
Feature importance using recursive feature elimination based on random forest.

- The correlation matrix was used to explore the relationships between the set of independent variables in the processed dataset and to decide on the list of independent variables that could be eliminated due to high correlation which may affect the model performance badly. The impact of multicollinearity is not an issue specific to regression models only, but it may affect classification models as well [47]. Its impact on classification models involves both the stability and the interpretability of the model [48]. In this study, the criteria that were followed in handling the multicollinearity issue between independent variables were based on eliminating one of the independent features that has a correlation coefficient value greater than 0.7 with another independent one [49]. Figure 13 shows the correlation matrix for a subset of independent features related to the medical history of participants and the raised blood sugar (RBS) target feature. In Figure 13, it can be seen there is a high collinearity between a set of independent features, represented in the relationship between prevalent hypertension and taking high blood pressure medication features, the second collinearity is found between prevalent diabetes and taking diabetes medication features, and the third one is between prevalent cholesterol and raised cholesterol medication features. All of these combinations have collinearity with a correlation coefficient value of 0.8, which is greater than the threshold of 0.7. In this step, the selected features to eliminate are those related to taking medication variables.
- The chi-square was used to identify the categorical independent set of variables that is correlated with the target variable of raised blood sugar. Figure 14 below shows the list of independent categorical features identified as the top 15 important features in predicting the target feature of raised blood sugar. This list included the history of cholesterol, history of hypertension, history of CVD, raised blood pressure, level of sugar intake, history of osteoporosis, physical inactivity, sleep disturbances, gender, former smoker, level of salt intake, current smoker, anxiety and depression (PHQ4), insufficiency of fruit and vegetable intake, and history of asthma, as the most important features identified by the chi-square test for the prediction of raised blood sugar. The history of diabetes was not forwarded to this test of feature importance since it is correlated with the outcome variable which can affect the predictive power of the model. This step helped in identifying the important predictor variables for the outcome features related to raised blood sugar and raised blood pressure prediction models. However, no variable was eliminated within this step, the set variables were forwarded for further phase of exploring features importance using the random forest classifier.
- A random forest classifier was utilized as the final step in the feature selection process. The random forest classifier was used to obtain the optimal feature selection process, by forwarding all types of variables, either categorical or continuous, as the input and identifying their performance in comparison to the outcome variables in the proposed models [50]. Utilizing the random forest in the variable’s selection process is considered one of the most powerful methods to determine the appropriate and significant features that contribute to the prediction of the outcome feature in machine learning models [51]. Figure 15 illustrates the results of feature importance that were obtained by integrating the random forest classifier in selecting the top 30 features for predicting the raised blood sugar outcome variable.

Based on the given results, the final list of features that will be used for the next stage of training the raised blood sugar prediction model are age, triglyceride, waist-to-hip ratio, BMI, waist circumference, heart rate, total cholesterol, hip circumference, HDL cholesterol, history of cholesterol, sugar intake level, history of hypertension, inadequate sleeping hours, insufficient physical activity, anxiety and depression, level of salt intake, history of CVD, raised blood pressure, passive smoking, mental health ill-being, JSS sleep disturbances, insufficient intake of fruits and vegetables, gender, smoking, history of osteoporosis, former smoker, history of asthma, history of cancer, history of renal failure, and alcohol consumption.

#### 3.4.3. Feature Scaling

The STEPS dataset contains numerical features from various scales, which can pose computational challenges during predictive modeling. Therefore, the min–max normalization technique was utilized to scale the numerical features, reducing computational complexity. This technique normalizes the features to a range between 0 and 1, as shown in Equation (1) [52].

(1) $$\mathrm{Xnorm}=\frac{X-\mathrm{Xmin}}{\mathrm{Xmax}-\mathrm{Xmin}},$$

#### 3.4.4. Dataset Oversampling

**Figure 16:**
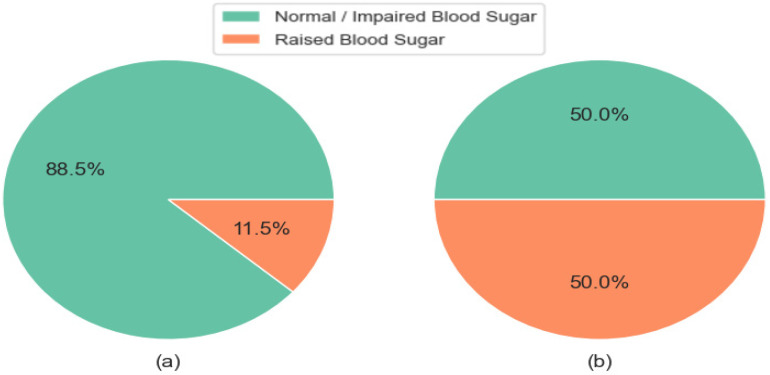
Distribution of cases by the level of blood sugar before (**a**) and after (**b**) applying oversampling.

Through exploratory data analysis of the processed dataset, it was identified that the target feature, indicating raised blood sugar level, exhibits class imbalance. Specifically, only 11.5% of observations in the dataset indicate raised blood sugar levels, while 88.5% signify normal blood sugar levels, as shown in Figure 16. This imbalance can adversely affect machine learning algorithms, as class imbalance is a significant challenge in classification algorithms [53]. To mitigate this issue, the oversampling technique was implemented [54]. This involved replicating observations from the minority class (raised blood sugar levels) to match the size of the majority class (not raised blood sugar levels).

### 3.5. Machine Learning Models

**Figure 17:**
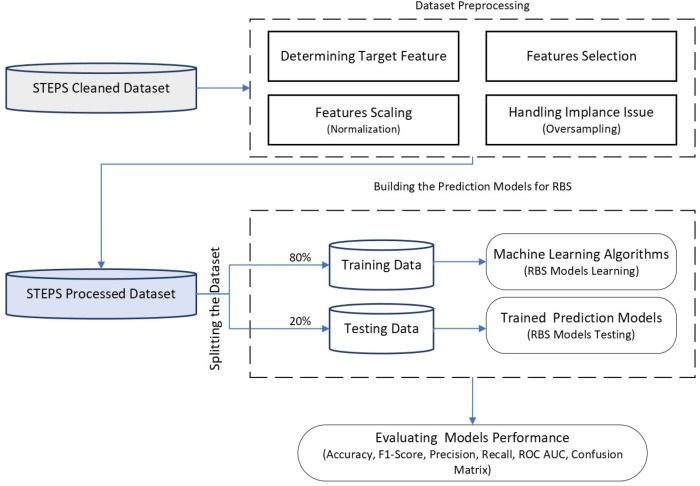
The workflow of the raised blood sugar detection models.

This section outlines the workflow of the proposed model and the machine learning algorithms utilized for building the raised blood sugar prediction models. Figure 17 illustrates the workflow of the proposed raised blood sugar (RBS) detection models.

Referring to Figure 2 in this section, Figure 17 below outlines the details of the last three phases and their components used to construct the proposed models. These three phases are closely linked, particularly for building machine learning models.

The workflow in Figure 17 highlights the core phases of the entire process, for constructing the proposed models from a machine learning perspective. This includes the essential data preprocessing phase before model training outlined in the previous Section 3.4, the training and testing of the constructed models presented in this section, and the final phase for performance evaluation outlined in the next section.

The first step in the modeling process is splitting the processed dataset into two subsets, one of them for training the models using 80% of observations from the entire processed dataset, and the remaining portion of 20% to be used later for testing the results of the trained models. Subsequently, after the phase of model learning is completed, the testing subset is passed into the trained models. Finally, using the predicted outcomes, the models’ performance measures are calculated to evaluate the effectiveness of the implemented models.

The raised blood sugar prediction models are implemented using a set of various powerful machine learning algorithms including random forest (RF), decision tree (DT), XGBoost, Adaboost, bagging decision trees, and multi-layer perceptron (MLP). The parameter settings for each algorithm employed in these models are configured using the GridSearch approach to set up the parameters that yield the best performance results.

- Decision tree is a supervised machine learning algorithm that can be utilized to build classification and regression models. It is marked as one of the simplest straightforward machine learning algorithms, and is based on arranging the features in a tree structure, and recursively splitting them based on chosen impurity criteria, such as entropy measure, Gini index, or information gain value [55].
- The AdaBoost algorithm which stands for adaptive boosting is a supervised machine learning algorithm used for building classification models, based on combining multiple weak classifiers to obtain more improved performance, the AdaBoost classifier commonly uses one level decision tree classifier. In AdaBoost, overfitting problems can be less likely to occur with it compared to other learning algorithms; however, it is not a suitable choice for datasets containing outliers and noisy data [56].
- Random forest is one of the most robust supervised machine learning algorithms commonly used for classification tasks. It is based on constructing a forest of multiple decision trees for a subset of the variables that are selected randomly in each tree. The prediction results of all the generated decision trees are aggregated to obtain the final refined output. By using this ensemble technique, the random forest makes an improved performance by mitigating the high variance issue that is known as a common issue in the decision tree [57].
- The XGBOOST algorithm, short for extreme gradient boosting, is a supervised machine learning tree-based algorithm that is an improved version of its earlier gradient boost algorithm. It can be applied for regression and classification problems, in particular for large datasets due to its high efficiency in generating accurate results and fast execution time. The working approach of the XGBOOST is based on passing the outcome of a processed tree into the next tree sequentially [58].
- Bagging decision trees, which stands for bootstrap aggregating, is a machine learning algorithm that improves the accuracy and stability of decision trees by using the ensemble approach. Bagging decision trees train multiple decision tree classifiers independently, with training each tree on a random sample of the training data with replacement (bootstrap sampling). Based on a different subset of data, each decision tree predicts the target variable, and their predictions are aggregated to determine the final outcome. For regression tasks, this aggregation is achieved by averaging the predictions, and for classification tasks by voting. Using the bagging technique reduces overfitting and variance, which improves the model’s overall performance and robustness [59].
- The multi-layer perceptron classifier (MLP) is a feedforward ANN algorithm used for classification problems. It consists of multiple layers of interconnected nodes associated with weights and uses an iterative optimization algorithm of backpropagation to minimize error and optimize classification results. The MLP classifier has the well-known advantage of handling the nonlinearity issue of relationships in the processed data [60].

### 3.6. Performance Evaluation Criteria

Various measures are employed to evaluate the models’ performance in predicting and detecting raised blood sugar cases. These metrics encompass accuracy, precision, recall, F1-score, confusion matrix, and ROC AUC, which are mainly calculated from fundamental measures, true positive (TP), false positive (FP), true negative (TN), and false negative (FN), which are used commonly to evaluate classification models [61].

- Confusion matrix: used commonly to summarize the prediction results of the machine learning classification models, by comparing the actual values versus predicted values, by which also the different performance metrics can be calculated. The confusion matrix consists of the following four measures:
- TP: the number of records from the positive class predicted correctly by the model.
- FP: the number of records from the negative class predicted incorrectly as a positive class by the model.
- TN: the number of records from the negative class predicted correctly by the model.
- FN: the number of records from the positive class predicted incorrectly as a negative class by the model.
- Accuracy: performance measure used to evaluate the efficiency of machine learning classification models. It is computed as the ratio of correct prediction to the total number of predictions.

(2) $$\mathrm{Accuracy}=\frac{\mathrm{TP}+\mathrm{TN}}{\mathrm{TP}+\mathrm{TN}+\mathrm{FP}+\mathrm{FN}},$$

- Precision is calculated as the fraction of correct prediction from the positive class to the total number of predictions as a positive class, in this model it represents the proportion of those who were predicted correctly as raised blood sugar cases, to the total number of observations predicted as raised blood sugar cases.

(3) $$\mathrm{Precision}=\frac{\mathrm{TP}}{\mathrm{TP}+\mathrm{FP}},$$

- Recall metric is the fraction of correctly predicted observations out of all actual observations of raised blood sugar cases.

(4) $$\mathrm{Recall}=\frac{\mathrm{TP}}{\mathrm{TP}+\mathrm{FN}},$$

- F1-Score is calculated as the harmonic mean of precision and recall metrics.

(5) $$F1\text{-}\mathrm{score}=2*\frac{(\mathrm{Precision}*\mathrm{Recall})}{\mathrm{Precision}+\mathrm{Recall}},$$

- The Receiver Operator Characteristic (ROC) is a visual representation that shows how well a machine learning model can differentiate between several classes, by plotting the true positive rate (TPR) and the false positive rate. ROC curve is a visual evaluation method for the performance of classification models, which works by calculating the area under the curve (AUC), the greater the AUC value the better the performance of the classification model [62].

(6) $$\mathrm{TPR}=\frac{\mathrm{TP}}{\mathrm{TP}+\mathrm{FN}},$$

(7) $$\mathrm{FPR}=\frac{\mathrm{FP}}{\mathrm{FP}+\mathrm{TN}}$$

## 4. Results and Discussion

This section discusses the experimental results of the raised blood sugar prediction models. The performance of the raised blood sugar detection and prediction models was evaluated using the metrics presented in Section 3.4. Table 2 presents the results of the performance evaluation using various measures including accuracy, recall, precision, and F1 Score. The random forest algorithm has the highest scores in accuracy, precision, and F1-Score among all examined models. The scores were 98.4%, 98.4%, and 97.1% for accuracy, F1-Score, and precision, respectively.

**Figure 18:**
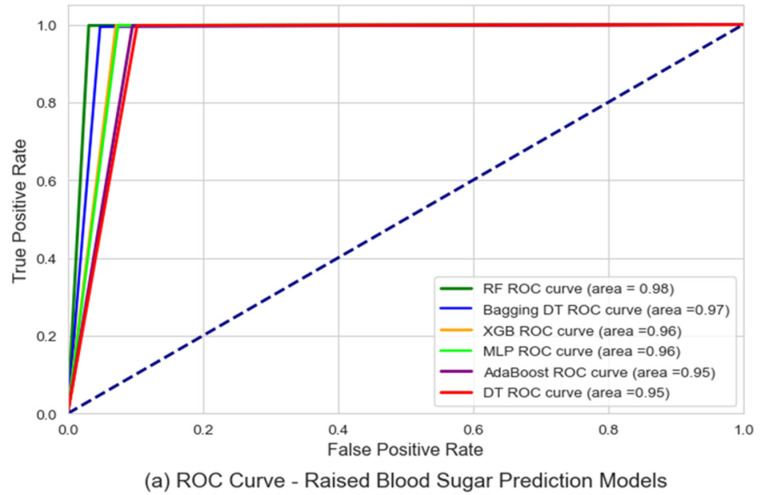

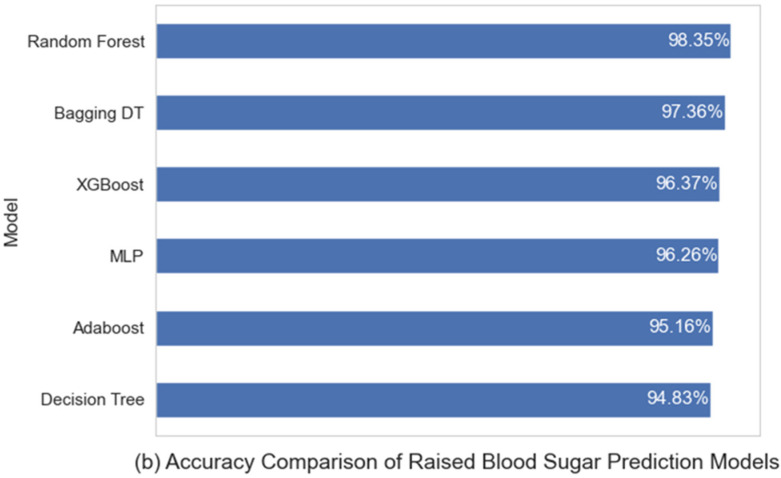
Raised blood sugar prediction models performances comparison using ROC AUC in (**a**), and accuracy in (**b**).

Figure 18a presents the testing results for the raised blood sugar detection and prediction models using the ROC AUC, represented by the ratio of negative observations (not raised blood sugar levels cases) that were predicted incorrectly as raised blood sugar levels out of the total number of observations from the negative class (specificity), versus the ratio of positive observations (raised blood sugar levels cases) that were predicted correctly as raised blood sugar levels cases out of the total number of observation from the positive class (sensitivity). Also, Figure 18b presents a comparison of the accuracy for all the utilized raised blood sugar detection and prediction models.

From the presented results it can be concluded that the random forest algorithm had the highest performance, achieving an AUC value of 0.98, and an accuracy of 98.4%, followed by the bagging decision trees model with an AUC value of 0.96, while the decision tree-based model had the lowest performance with an AUC of 0.95 and accuracy of 94.8%.

The highest performance result for the raised blood sugar prediction model which was implemented using the random forest classifier might be attributed to the ensemble working approach in random forest, minimizing the high variance issues that are present in the decision tree, as well as to its low sensitivity to overfitting problems. On the other hand, the model that was utilized using the multi-layer perceptron algorithm which is based on neural networks did not yield the best performance results, although neural network algorithms are well known for their high predictive power and robustness; however, this can vary depending on the characteristics and complexity of the used dataset, in addition to the fact that using MLP ANN requires very careful configuration of the algorithm parameters. For models using the Adaboost and XGBoost algorithms it is noticed that their performance is higher than the decision tree-based model, this result aligns with the basics of these algorithms, of using the decision tree as a weak learning and improving its performance by aggregating multiple weak predictions to reduce bias and high variance issues.

**Figure 19:**
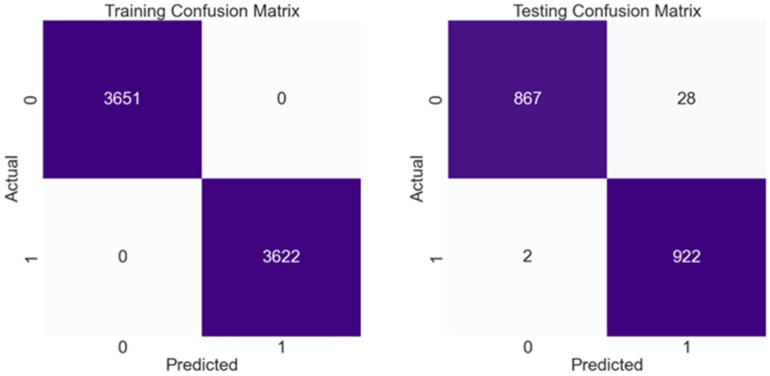
Comparison of the training and testing performance of random forest model using confusion matrix.

The confusion matrix in Figure 19 summarizes the performance of the raised blood sugar prediction model, which is implemented using the random forest classifier, which achieved the highest performance among the tested models, showing the correct and incorrect predictions from the positive and negative classes related to raised blood sugar subject, and not raised blood sugar subject, respectively.

Based on the previous results, it can be concluded that the proposed model for predicting raised blood sugar in this study outperforms the results achieved in other models implemented in previous studies that were applied to similar datasets of risk factors related to diabetes. In addition, the accuracy and other performance metrics are significantly higher in this model, despite it being applied to a larger dataset including all possible variables that are found to contribute to the development of diabetes and other NCDs.

Analysis of the features’ importance in the proposed model for raised blood sugar prediction shows that age, gender, obesity, waist–hip ratio, total cholesterol, HDL, hypertension, history of cardiovascular diseases, physical activity, sugar intake, salt intake, anxiety and depression, and sleep disturbances variables contribute substantially towards the predictive power of the model.

## 5. Conclusions

This study proposes machine learning-based models to detect and predict raised blood sugar levels, using a curated selection of robust supervised machine learning classification algorithms. These algorithms leverage various risk factors present in the dataset. The models are built using sex primary classification algorithms: random forest, decision tree, Adaboost, XGBoost, and bagging decision trees, in addition to using multi-layer perceptron from neural networks. Among these, the random forest classifier achieved the highest accuracy rate of 98.4%. There were also promising results for bagging decision trees, XGBoost, MLP, AdaBoost, and decision tree, achieving accuracy rates of 97.4%, 96.4%, 96.3%, 95.2%, and 94.8%, respectively. The significance of using these models lies in their potential to support the healthcare sector and alleviate the workload of healthcare workers. This will contribute to improved public health and well-being. For future endeavors, it is recommended to incorporate more than one reading for the fasting blood sugar for the use of diagnosis purposes. Further, the model can also be enhanced to determine whether fasting blood sugar is normal, impaired, or high. Another potential opportunity for improving the study results could be achieved by collecting datasets from the STEPS NCDs studies that were conducted in different communities and countries for performing model generalization by training it on datasets collected from various ethnicities.

Finally, deploying the raised blood sugar prediction model into health management information systems and electronic health records presents several challenges. These include privacy limitations in acquiring consent for using patients’ data to build predictive models and the acceptance of incorporating machine learning and artificial intelligence techniques within the healthcare sector among the public, patients, and healthcare professionals. Additionally, the availability of electronic health records, especially in developing countries, poses a significant limitation, as many healthcare facilities still rely on paper-based and manual approaches for reporting and documenting health data. In addition, researchers should avoid complicated and numerous predictors and focus on simple and accurate predictors.

## Figures and Tables

**Table 1:** Research findings related to machine learning prediction model for glucose metabolism disorders.

Ref.	Dataset	Features	Target Feature	Algorithms	Best Model	Outcome %
[25]	PIDD	age, number of pregnancies, glucose, diabetes pedigree function, blood pressure, skin thickness, insulin, BMI	Diabetic class (yes/no)	ANN, RF, K-means	ANN	Accuracy 75.7
[27]	PIDD	number of pregnancies, glucose, blood pressure, skin thickness, insulin, BMI, diabetes pedigree function, age	Diabetic class (yes/no)	SVM, DT, KNN, RF, AdaBoost. NB, LR, ANN	ANN	Accuracy 88.6
[28]	UCI Diabetes Dataset	age, gender, polyuria, polydipsia, sudden weight loss, weakness, polyphagia, genital thrush, visual blurring, itching, irritability, delayed healing, partial paresis, muscle stiffness, alopecia, obesity	Diabetes patient (yes/no)	RF, SVM, KNN, and DT J48	DT J48(with noisy data) RF (without noisy data)	Accuracy 73.82 Accuracy 100.0
[30]	PIDD	number of pregnancies, glucose, blood pressure, skin thickness, insulin, BMI, diabetes pedigree function, age	Diabetic class (yes/no)	RF, SVM, CNN	RF	Accuracy 83.6
[31]	PIDD	number of pregnancies, glucose, blood pressure, skin thickness, insulin, BMI, diabetes pedigree function, age	Diabetic class (yes/no)	NB, DT, ANN, DL	DL	Accuracy 98.07
[32]	PIDD	number of pregnancies, glucose, blood pressure, skin thickness, insulin, BMI, diabetes pedigree function, age	Diabetic class (yes/no)	K-means and LR	Hybrid model (K-means and LR)	Accuracy 93.9
[33]	Privately collected dataset	age, gender, BMI, family history of diabetes, marital status, education level, stress, sleep, physical activity, diet, in-salt taking, and drinking coffee	Reported diabetes diagnosis (yes/no)	ANN, DT, LR	DT	Accuracy 77.87 Sensitivity 80.68 specificity 75.13
[33]	NHANES	age, waist size, leg length, sodium, fiber, caffeine intake, ethnicity and income	Reported diabetes diagnosis (yes/no)	LR, SVM, RF, XGBoost, Ensemble	XGBoost	ROC AUC 86.2 Precision, Recall, F1-score 78.0
[33]	NHANES	age, waist size, leg length, sodium, fiber, caffeine intake, ethnicity and income, HDL, LDL, cholesterol, urine	Reported diabetes diagnosis (yes/no)	LR, SVM, RF, XGBoost, Ensemble	XGBoost	ROC AUC 95.7 Precision, Recall, F1-score 89.0
[33]	NHANES	age, waist size, leg length, sodium, fiber, caffeine intake, ethnicity, and income	FBS ≥ 126 (yes/no)	LR, SVM, RF, XGBoost, Ensemble	Ensemble	ROC AUC 73.7 Precision, Recall, F1-score 68.0
[34]	NHANES	age, waist size, leg length, sodium, fiber, caffeine intake, ethnicity and income, HDL, LDL, cholesterol, urine	FBS ≥ 126 (yes/no)	LR, SVM, RF, XGBoost, Ensemble	XGBoost	ROC AUC 80.2 Precision, Recall, F1-score 68.0
[36]	NHANES	BMI, family history of diabetes, race, hypertension, cholesterol	FBS ≥ 100, or 2hrPG ≥ 140, or HbA1C ≥ 5.7% (yes/no)	RF, AdaBoost, LR, J48, NB, PART, SMO, IBk, LogitBoost	NB	Accuracy 74.5
[37]	Privately collected dataset	age, sex, BMI, blood pressure, triglyceride, HDL, LDL, creatinine, total cholesterol, FBS, HbA1C, IRI, PG	FBS ≥ 100, or 2hrPG ≥ 140, or HbA1C ≥ 5.7% (yes/no)	LR, XGBoost	XGBoost	ROC AUC 78.0

**Table 2:** The results of performance metrics for the raised blood sugar prediction models.

Algorithm	RF	Bagging DT	MLP	XGBoost	AdaBoost	DT
Accuracy	98.4%	97.4%	96.3%	96.4%	95.2%	94.8%
F1-Score	98.4%	97.5%	96.5%	96.5%	95.4%	95.2%
Precision	97.1%	95.3%	93.2%	93.5%	91.5%	90.9%
Recall	99.8%	99.5%	99.9%	99.8%	99.8%	99.8%

## Data Availability

The data and source code used in this paper can be shared with other researchers upon a reasonable request.

## References

[1] The World Health Organization Diabetes. https://www.who.int/news-room/fact-sheets/detail/diabetes.

[2] Clark N.G., Fox K.M., Grandy S. (2007). Symptoms of diabetes and their association with the risk and presence of diabetes: Findings from the study to help improve early evaluation and management of risk factors leading to diabetes (SHIELD). Diabetes Care.

[3] Forouhi N.G., Wareham N.J. (2010). Epidemiology of diabetes. Medicine.

[4] Zheng Y., Ley S.H., Hu F.B. (2017). Global aetiology and epidemiology of type 2 diabetes mellitus and its complications. Nat. Rev. Endocrinol..

[5] Soomro M.H., Jabbar A. (2024). Diabetes etiopathology, classification, diagnosis, and epidemiology. BIDE’s Diabetes Desk Book.

[6] IDF Diabetes Atlas 2021|IDF Diabetes Atlas. https://diabetesatlas.org/atlas/tenth-edition/.

[7] Bloomgarden Z., Handelsman Y. (2023). Diabetes Epidemiology and Its Implications. Lipoproteins in Diabetes Mellitus.

[8] American Diabetes Association Professional Practice Committee (2024). 12. Retinopathy, Neuropathy, and Foot Care: Standards of Care in Diabetes—2024. Diabetes Care.

[9] Alqadi S.F. (2024). Diabetes Mellitus and Its Influence on Oral Health: Review. Diabetes Metab. Syndr. Obes..

[10] Williams R., Airey M. (2002). Epidemiology and Public Health Consequences of Diabetes. Curr. Med. Res. Opin..

[11] The World Health Organization The Top 10 Causes of Death. https://www.who.int/news-room/fact-sheets/detail/the-top-10-causes-of-death.

[12] Laine C., Caro J.F. (1996). Preventing complications in diabetes mellitus: The role of the primary care physician. Med. Clin. N. Am..

[13] Tiwary N., Sharma N., Singh S., Behl T., Zahoor I. (2023). Understanding the Pharmacological and Nanotechnological Facets of Dipeptidyl Peptidase-4 Inhibitors in Type II Diabetes Mellitus: A Paradigm in Therapeutics. Bionanoscience.

[14] American Diabetes Association (2020). 2. Classification and Diagnosis of Diabetes: Standards of Medical Care in Diabetes—2020. Diabetes Care.

[15] Peng W.K., Chen L., Boehm B.O., Han J., Loh T.P. (2020). Molecular phenotyping of oxidative stress in diabetes mellitus with point-of-care NMR system. NPJ Aging Mech. Dis..

[16] The World Health Organization Mean Fasting Blood Glucose. https://www.who.int/data/gho/indicator-metadata-registry/imr-details/2380.

[17] Owess M.M., Owda A.Y., Owda M. Decision Support System in Healthcare for Predicting Blood Pressure Disorders. Proceedings of the 2023 International Conference on Information Technology: Cybersecurity Challenges for Sustainable Cities, ICIT 2023—Proceeding.

[18] Saleem T.J., Chishti M.A. (2019). Exploring the Applications of Machine Learning in Healthcare. Int. J. Sens. Wirel. Commun. Control..

[19] Singh P., Singh N., Singh K.K., Singh A. (2021). Diagnosing of disease using machine learning. Machine Learning and the Internet of Medical Things in Healthcare.

[20] Jaiswal V., Negi A., Pal T. (2021). A review on current advances in machine learning based diabetes prediction. Prim. Care Diabetes.

[21] Zhu T., Li K., Herrero P., Georgiou P. (2021). Deep Learning for Diabetes: A Systematic Review. IEEE J. Biomed. Health Inform..

[22] Varma K.M., Panda B.S. (2019). Comparative analysis of Predicting Diabetes Using Machine Learning Techniques. J. Emerg. Technol. Innov. Res..

[23] Makalesi A., Nur Ergün Ö., İlhan H.O. (2021). Early Stage Diabetes Prediction Using Machine Learning Methods. Avrupa Bilim Teknol. Derg..

[24] Islam M.T., Al-Absi H.R.H., Ruagh E.A., Alam T. (2021). DiaNet: A Deep Learning Based Architecture to Diagnose Diabetes Using Retinal Images only. IEEE Access.

[25] Mahboob Alam T., Iqbal M.A., Ali Y., Wahab A., Ijaz S., Baig T.I., Hussain A., Malik M.A., Raza M.M., Ibrar S. (2019). A model for early prediction of diabetes. Inform. Med. Unlocked.

[26] UCI Machine Learning and Kaggle, Pima Indians Diabetes Database. https://www.kaggle.com/datasets/uciml/pima-indians-diabetes-database/data.

[27] Khanam J.J., Foo S.Y. (2021). A comparison of machine learning algorithms for diabetes prediction. ICT Express.

[28] Kandhasamy J.P., Balamurali S. (2015). Performance Analysis of Classifier Models to Predict Diabetes Mellitus. Procedia Comput. Sci..

[29] Aitbayev A. Diabetes UCI Dataset. https://www.kaggle.com/datasets/alakaaay/diabetes-uci-dataset.

[30] Yahyaoui A., Jamil A., Rasheed J., Yesiltepe M. A Decision Support System for Diabetes Prediction Using Machine Learning and Deep Learning Techniques. Proceedings of the 1st International Informatics and Software Engineering Conference: Innovative Technologies for Digital Transformation, IISEC 2019—Proceedings.

[31] Naz H., Ahuja S. (2020). Deep learning approach for diabetes prediction using PIMA Indian dataset. J. Diabetes Metab. Disord..

[32] Wu H., Yang S., Huang Z., He J., Wang X. (2018). Type 2 diabetes mellitus prediction model based on data mining. Inform. Med. Unlocked.

[33] Meng X.H., Huang Y.X., Rao D.P., Zhang Q., Liu Q. (2013). Comparison of three data mining models for predicting diabetes or prediabetes by risk factors. Kaohsiung J. Med. Sci..

[34] Dinh A., Miertschin S., Young A., Mohanty S.D. (2019). A data-driven approach to predicting diabetes and cardiovascular disease with machine learning. BMC Med. Inform. Decis. Mak..

[35] Centers for Disease Control and Prevention, NHANES Questionnaires, Datasets, and Related Documentation. https://wwwn.cdc.gov/nchs/nhanes/Default.aspx.

[36] Vangeepuram N., Liu B., Chiu P.H., Wang L., Pandey G. (2021). Predicting youth diabetes risk using NHANES data and machine learning. Sci. Rep..

[37] Maeta K., Nishiyama Y., Fujibayashi K., Gunji T., Sasabe N., Iijima K., Naito T. (2018). Prediction of Glucose Metabolism Disorder Risk Using a Machine Learning Algorithm: Pilot Study. JMIR Diabetes.

[38] Noncommunicable Disease Surveillance, Monitoring and Reporting. https://www.who.int/teams/noncommunicable-diseases/surveillance/systems-tools/steps.

[39] Owda M., Owda A.Y., Fasli M. An Exploratory Data Analysis and Visualizations of Underprivileged Communities Diabetes Dataset for Public Good. Proceedings of the 2023 22nd IEEE/WIC International Conference on Web Intelligence and Intelligent Agent Technology, WI-IAT 2023.

[40] Ferrannini E., Cushman W.C. (2012). Diabetes and hypertension: The bad companions. Lancet.

[41] De Boer I.H., Bangalore S., Benetos A., Davis A.M., Michos E.D., Muntner P., Rossing P., Zoungas S., Bakris G. (2017). Diabetes and hypertension: A position statement by the American diabetes association. Diabetes Care.

[42] Nguyen N.T., Magno C.P., Lane K.T., Hinojosa M.W., Lane J.S. (2008). Association of Hypertension, Diabetes, Dyslipidemia, and Metabolic Syndrome with Obesity: Findings from the National Health and Nutrition Examination Survey, 1999 to 2004. J. Am. Coll. Surg..

[43] Jafar T.H., Chaturvedi N., Pappas G. (2006). Prevalence of overweight and obesity and their association with hypertension and diabetes mellitus in an Indo-Asian population. Cmaj.

[44] Abdullah A., Peeters A., de Courten M., Stoelwinder J. (2010). The magnitude of association between overweight and obesity and the risk of diabetes: A meta-analysis of prospective cohort studies. Diabetes Res. Clin. Pract..

[45] Amarnath B., Balamurugan S., Alias A. (2016). Review on feature selection techniques and its impact for effective data classification using UCI machine learning repository dataset. J. Eng. Sci. Technol..

[46] Chen R.C., Dewi C., Huang S.W., Caraka R.E. (2020). Selecting critical features for data classification based on machine learning methods. J. Big Data.

[47] Misra P., Yadav A.S. (2020). Improving the classification accuracy using recursive feature elimination with cross-validation. Int. J. Emerg. Technol..

[48] Drobnič F., Kos A., Pustišek M. (2020). On the interpretability of machine learning models and experimental feature selection in case of multicollinear data. Electronics.

[49] Dormann C.F., Elith J., Bacher S., Buchmann C., Carl G., Carré G., Marquéz J.R.G., Gruber B., Lafourcade B., Leitão P.J. (2013). Collinearity: A review of methods to deal with it and a simulation study evaluating their performance. Ecography.

[50] Reif D.M., Motsinger A.A., McKinney B.A., Crowe J.E., Moore J.H. Feature selection using a random forests classifier for the integrated analysis of multiple data types. Proceedings of the 2006 IEEE Symposium on Computational Intelligence in Bioinformatics and Computational Biology, CIBCB’06.

[51] Khan N.M., Madhav C.N., Negi A., Thaseen I.S. (2020). Analysis on Improving the Performance of Machine Learning Models Using Feature Selection Technique. Advances in Intelligent Systems and Computing.

[52] Raju V.N.G., Lakshmi K.P., Jain V.M., Kalidindi A., Padma V. Study the Influence of Normalization/Transformation process on the Accuracy of Supervised Classification. Proceedings of the 3rd International Conference on Smart Systems and Inventive Technology, ICSSIT 2020.

[53] Cecchini V., Nguyen T.P., Pfau T., De Landtsheer S., Sauter T. An efficient machine learning method to solve imbalanced data in metabolic disease prediction. Proceedings of the 2019 11th International Conference on Knowledge and Systems Engineering, KSE 2019.

[54] Gosain A., Sardana S. Handling class imbalance problem using oversampling techniques: A review. Proceedings of the 2017 International Conference on Advances in Computing, Communications and Informatics, ICACCI 2017.

[55] Sharma H., Kumar S. (2016). A Survey on Decision Tree Algorithms of Classification in Data Mining. Int. J. Sci. Res..

[56] Cao Y., Miao Q.-G., Liu J.-C., Gao L. (2013). Advance and Prospects of AdaBoost Algorithm. Acta Autom. Sin..

[57] Ziegler A., König I.R. (2014). Mining data with random forests: Current options for real-world applications. Wiley Interdiscip Rev. Data Min. Knowl. Discov..

[58] Chen T., Guestrin C. XGBoost: A scalable tree boosting system. Proceedings of the ACM SIGKDD International Conference on Knowledge Discovery and Data Mining.

[59] Abellán J., Masegosa A.R. (2010). Bagging decision trees on data sets with classification noise. Lecture Notes in Computer Science.

[60] Fiesler E., Beale R. (2020). Multilayer perceptrons. Handbook of Neural Computation.

[61] Dj Novakovi J., Veljovi A., Ili S.S., Papi Ž., Tomovi M. (2017). Evaluation of Classification Models in Machine Learning. Theory Appl. Math. Comput. Sci..

[62] Sø K. (2009). Receiver-operating characteristic curve analysis in diagnostic, prognostic and predictive biomarker research. J. Clin. Pathol..

